# Exposure of chlorothalonil and acetamiprid reduce the survival and cause multiple internal disturbances in *Apis mellifera* larvae reared *in vitro*

**DOI:** 10.3389/fphys.2023.1114403

**Published:** 2023-02-13

**Authors:** Ying Lu, Jing Gao, Tong Wu, Bo Han, Bingnan Qian, Min Shi, Sa Yang, Qingyun Diao, Chunya Bu, Pingli Dai

**Affiliations:** ^1^ Key Laboratory of Northern Urban Agriculture of Ministry of Agriculture and Rural Affairs, College of Bioscience and Resource Environment, Beijing University of Agriculture, Beijing, China; ^2^ Key Laboratory of Pollinating Insect Biology of Agriculture, Institute of Apicultural Research, Chinese Academy of Agricultural Sciences, Beijing, China

**Keywords:** honey bees, chlorothalonil, acetamiprid, toxicity, oxidative stress

## Abstract

**Background:** Chlorothalonil and acetamiprid are chemical pesticides commonly used in agricultural production and have been shown to have negative effects on bee’s fitness. Despite many studies have revealed that honey bee (*Apis mellifera* L.) larvae are posting a high risk on exposure to pesticides, but the toxicology information of chlorothalonil and acetamiprid on bee larvae remain limited.

**Results:** The no observed adverse effect concentration (NOAEC) of chlorothalonil and acetamiprid for honey bee larvae were 4 μg/mL and 2 μg/mL, respectively. Except for CarE, the enzymic activities of GST and P450 were not influenced by chlorothalonil at NOAEC, while chronic exposure to acetamiprid slightly increased the activities of the three tested enzymes at NOAEC. Further, the exposed larvae showed significantly higher expression of genes involved in a series of different toxicologically relevant process following, including caste development (*Tor* (GB44905), *InR-2* (GB55425), *Hr4* (GB47037), *Ac3* (GB11637) and *ILP-2* (GB10174)), immune system response (*abaecin* (GB18323), *defensin-1* (GB19392), *toll-X4* (GB50418)), and oxidative stress response (P450, GSH, GST, CarE).

**Conclusion:** Our results suggest that the exposure to chlorothalonil and acetamiprid, even at concentrations below the NOAEC, showed potentially effects on bee larvae’s fitness, and more important synergistic and behavioral effects that can affect larvae fitness should be explored in the further.

## Introduction

Over the last few decades, significant declines in insect populations and diversity in several regions of the globe have been alarming, especially for bee species (El-Seedi et al., 2022). Although multiple factors likely contribute to the observed bee declines, e.g., the parasitic mite, viruses, pollution and climate change (Human et al., 2014; Myers et al., 2017; Dolezal et al., 2019; Traynor et al., 2020), an essential reason is excessive pesticide use of agrochemicals, which has been proved to lead to impairment of essential functions, such as reproduction, foraging and homing, thereby affecting overall health and population of the bee colony (Wang et al., 2020; Wagner et al., 2021). Thus, much attention has been directed toward the safety of pesticides to bees.

Compared to adults, *Apis mellifera* (honey bee) larvae are more susceptible to long-term exposure to sublethal doses of pesticides (Desneux et al., 2007). During flowering period, pollen and nectar with pesticide residues collected by foragers will be processed by the nurse bees and fed to developing larvae, providing potential opportunities for bee larvae to be exposed to pesticides during whole pre-brood stage (Mullin et al., 2010; Dai et al., 2018; Quintana et al., 2019). Moreover, high levels of pesticides were detected in beeswax from the brood nest where larvae develop (Medici et al., 2012; McAfee et al., 2021). Despite indirectly exposed to pesticides, larvae are usually less tolerant to pesticides than adults (Tan et al., 2017; Dai et al., 2018; O’neal et al., 2019; Tavares et al., 2019), nevertheless, the toxicology information on honey bee larvae are rare at present.

Pesticides have a range of sublethal effects on larvae of honey bee. Previous studies have revealed that chronic exposure to pesticides during the larval period can disrupt the normal growth and development of honey bee larvae, leading to malformations, reduced body size, and delayed development (Wu et al., 2011; Tan et al., 2015; Rosa et al., 2016). Long-term exposure to pesticides at early life stages can damage the nervous system of honey bee larvae, leading to behavioral abnormalities and reduced cognitive function as adult individuals (Tan et al., 2017). In addition, pesticides can weaken the immune and defense system of honey bee larvae, making them more susceptible to diseases and parasites (Tarek et al., 2018). For instance, exposure to sublethal doses of thiacloprid significantly aggravated the proliferation of black queen cell virus (BQCV) on host larval and boost the harmful effects of the virus on honey bees (Doublet et al., 2015). Sublethal pesticide exposure in honey bees larvae can cause oxidative stress by increasing the levels of reactive oxygen species (ROS) in the bees’ bodies, which can cause damage to cellular components such as DNA, proteins, and lipids (Prezenska et al., 2019; Yu et al., 2021). The GST (glutathione S-transferase) gene family encodes enzymes that participates in the process of ROS removal by interaction with glutathione (Schultzhaus et al., 2017). Catalase (CAT) and superoxide dismutase (SOD) are important enzymes establishing the first line of antioxidant defense systems. Previous studies have demonstrated that when exposed to low doses of pesticides, honey bee larvae are able to neutralize harmful oxidative compounds produced by oxidative stress *via* upregulating the expression of these enzymes or elevating their activities (du Rand et al., 2017; Yu et al., 2021).

Fungicides and neonicotinoid insecticides are common xenobiotics detected in bee products (Pareja et al., 2011; Bridi et al., 2018). Chlorothalonil (2,4,5,6-tetrachloroisophthalonitrile) is one of the most popular broad-spectrum protective fungicide in the world (Wang et al., 2021). Chlorothalonil is commonly applied to flowering crops, thus providing a possible route of exposure for bees. Mullin et al. (2010) have reported a maximum residue value of 99 mg a.i./kg for chlorothalonil detected in pollen (Mullin et al., 2010). Although not acutely toxic to bees, several studies have identified potential sublethal effects, especially on larvae (Zhu et al., 2014; Dai et al., 2018; O’neal et al., 2019). As a fungicide, chlorothalonil may affect the fungal community in the honey bee gut. Our previous work showed that chlorothalonil significantly decreased the survival rate of immature bees and altered the gut microbiota of newly emerged honeybees when exposure concentrations were higher than 2 μg/mL (Wu et al., 2022). Acetamiprid is a representative of the first generation of neonicotinoids with broad-spectrum characteristic, widely used to control destructive agricultural pests (Shi et al., 2019). Given their low toxicity to mammalian and non-target pollinators, acetamiprid has become the key available neonicotinoid pesticides throughout the world (Wang et al., 2017; Grassl et al., 2018). It has been shown that sublethal acetamiprid doses had a negative effect on the learning and memory ability of adult bees (Wen et al., 2017; Chen et al., 2020). Chronic exposure to acetamiprid was found to have an effect on the expression of genes related to immune, detoxification, and memory in larvae and adults (Shi et al., 2020).

The goal of this study was to investigate the effects of chronic exposure to the chlorothalonil and acetamiprid on *A. mellifera* larvae. Through the *in vitro* larval rearing method, we measure the no observed adverse effect concentration (NOAEC) of larval bees. Furthermore, we evaluate the sublethal effects of chlorothalonil and acetamiprid on humoral immunity and biochemical markers of exogenous substance, as well as gene expression involved in honey bee caste development. This work will provide additional information on the risks of chlorothalonil and acetamiprid exposure to honey bee larvae, and ultimately help to determine the integrated pest management strategies that minimize the harm of pesticides to honey bees.

## Materials and methods

### Honey bees

Larvae source colonies were kept in the Institute of Apicultural Research, Chinese Academy of Agricultural Sciences (40°01′23′′N, 116°21′24′′E). The honey bee larvae were transferred to the laboratory and reared in incubator (Ningbo Haishu Saifu Experimental Instrument Factory) (rearing temperature 35°C ± 0.5°C, relative humidity 95% ± 5%).

### Chronic toxicity

The experiment consisted of 13 treatments, including chlorothalonil (purity 99.7% purchased from Sigma-Aldrich, Shanghai, China) at concentrations of 1, 2, 4, 8 and 16 μg/mL; acetamiprid (purity 99.9% purchased from BePure, Shanghai, China) at concentrations of 0.5, 1, 2, 4 and 8 μg/mL; a negative control; a solvent control (0.1% acetone) and a positive control (dimethoate purity (99.3% purchased from Sigma-Aldrich, Shanghai, China) 45 μg/mL). Each treatment was repeated three times. Larvae were taken from three different colonies, a single colony per replicate. *A. mellifera* larvae were reared *in vitro* as described by Yang et al. (2020). We obtained excessive 1-day-old larvae and fed 20 μL normal diet on D1. On the third day, 12 healthy larvae were selected from each replicate and fed with a 20 μL diet that contained different dilutions of chlorothalonil and acetamiprid. Each larva was respectively fed with 30, 40, 50 μL treated diet on D4, D5 and D6.

### Preparation of cDNA and qRT-PCR

On day 7, the *A. mellifera* larvae were collected and frozen in liquid nitrogen, then stored at −8 °C. RNA was extracted from larvae using TRIzol reagent. RNA quality was checked using the BioSpectrometer^®^ kinetic (Eppendorf, America). An OD_260_/_280_ ratio of RNA between 1.8 and 2.0 is required to meet the criteria. Then, using PrimeScript™ RT reagent Kit with gDNA Eraser (Takara, Japan), cDNA was obtained from 1.0 μg RNA. The RT-qPCR was performed as follows: 30 s at 95°C, followed by 40 cycles of 5 s at 95°C, then 30 s at 60°C. RT-qPCR was carried in triplicate. Table 1 summarizes the primers information and uses the house keeping gene β-actin as the control. The relative expression of the test gene was calculated using the method reported by Qi et al. (2020).

**TABLE 1:** The primers used in qRT-PCR.

Primer	Direction	Sequence 5’-3’	Gene bank
CYP9Q2	Forward	GAT​TAT​CGC​CTA​TTA​TTA​CTG	GB17793
	Reverse	GTTCTCCTTCCCTCTGAT	
CYP9Q3	Forward	GTT​CCG​GGA​AAA​TGA​CTA​C	GB19967
	Reverse	GGTCAAAATGGTGGTGAC	
Gtpx1	Forward	CGA​CAA​CTA​TAA​GGA​AGC​GAA​A	GB47478
	Reverse	AGA​TAG​AAA​AAC​GTC​TTC​GCC​T	
GSH	Forward	CAC​CAT​ATG​CAT​GGC​AAG​T	GB41663
	Reverse	TTGTTGTAGGCATCGCG	
abaecin	Forward	AGA​TCT​GCA​CAC​TCG​AGG​TCT​G	GB18323
	Reverse	TCG​GAT​TGA​ATG​GTC​CCT​GA	
Toll-X4	Forward	TAG​AGT​GGC​GCA​TTG​TCA​AG	GB50418
	Reverse	ATC​GCA​ATT​TGT​CCC​AAA​AC	
defensin-1	Forward	TGC​GCT​GCT​AAC​TGT​CTC​AG	GB19392
	Reverse	AAT​GGC​ACT​TAA​CCG​AAA​CG	
Ac3	Forward	GCAGAGGCTGAGGAAGGA	GB11637
	Reverse	AAT​GGC​ACT​TAA​CCG​AAA​CG	
ILP-2	Forward	TGCCAGTAGCAGAAGTAG	GB10174
	Reverse	TGACAAAGTTCGACCACA	
Tor	Forward	ACG​GGA​CGT​GAT​TTC​TCT​CA	GB44905
	Reverse	ACC​AAA​AGG​GAC​ACC​ATC​CA	
Hr4	Forward	ACA​CGG​TAA​GCA​GTT​CGA​GG	GB47037
	Reverse	CAG​CTC​GTC​CAA​GTT​CCT​CA	
InR-2	Forward	GGG​AAG​AAC​ATC​GTG​AAG​GA	GB55425
	Reverse	CAT​CAC​GAG​CAG​CGT​GTA​CT	
β-actin	Forward	CACTATACGCTTCTGGAC	GB17681
	Reverse	CTT​TCT​GTA​AGG​ATC​TTC​ATG	

### Activity of detoxification enzymes

All of honey bee larvae were collected and quickly placed in liquid nitrogen and stored at −80°C on D7. Homogenize each larval sample independently on ice in pre-chilled pH 7.4 PBS, then Centrifuge at 3,500 rpm for 20 min at 4°C. The protein concentration in the supernatant was determined by using the BCA Protein Assay Kit (Thermo Fisher Scientific, America). The P450, GST and CarE enzyme activities of the supernatant were determined using the ELISA assay kit from Shanghai Mlbio, China.

### Statistics

All data are expressed as the mean ± SE. The JMP 13 software produced the Kaplan-Meier curve. GraphPad Prism 9.0 were performed for One-way ANOVA on enzyme activity and gene expression levels. Tukey’s test (*p* = 0.05) was performed to determine the difference between every treatment and solvent control.

## Results

### Chronic toxicity of acetamiprid and chlorothalonil to honey bee larvae

**FIGURE 1:**
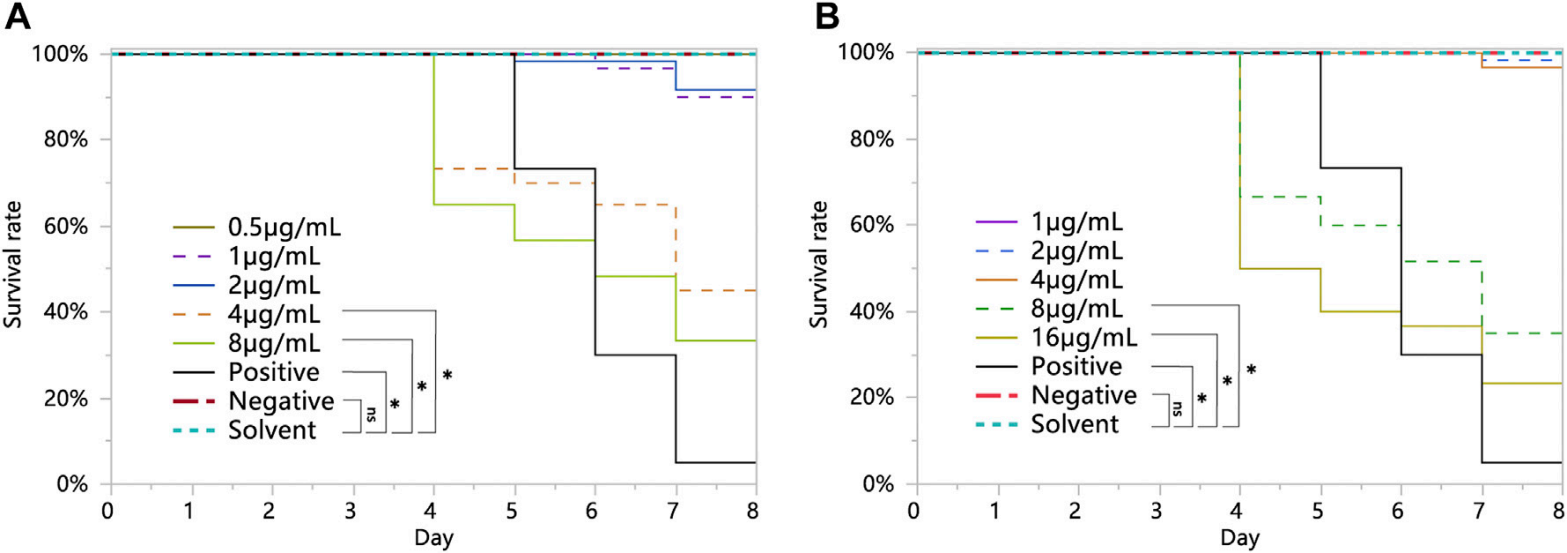
Overall survival of *Apis mellifera* larvae exposed to sublethal concentrations of chlorothalonil **(A)**, acetamiprid **(B)**, during larval development on D3 through D6 after grafting (*n* = 3 replicates of 12 larvae/replicate, or 36 larvae, per test substance). Larvae were fed a dimethoate-contaminated diet (45 mg/L) as a positive control, an acetone-contaminated diet as a solvent control. Significant differences were set at * for *p* < 0.05.

The survival of larvae fed with 8 and 16 μg/mL chlorothalonil was significantly lower than that of larvae fed the negative and solvent control diets (Figure 1A). However, the survival of larvae fed with 1, 2 and 4 μg/mL chlorothalonil was not significantly different from that of larvae fed with the solvent control and the negative control diets. The NOAEC of chlorothalonil to honey bee larvae was 4 μg/mL. The survival of larvae fed with 4 and 8 μg/mL acetamiprid was significantly lower than that of larvae fed the negative and solvent control diets (Figure 1B). However, the survival of larvae fed with 0.5, 1 and 2 μg/mL acetamiprid was not significantly different from that of larvae fed with the solvent control and the negative control diets. The NOAEC of acetamiprid to honey bee larvae was 2 μg/mL.

### Influence of acetamiprid and chlorothalonil on the expression of division related genes

Through statistical analysis, we found that survival rate had no significant difference between negative controls and solvent controls in our research. Thus, only solvent control was used for the following analysis.

**FIGURE 2:**
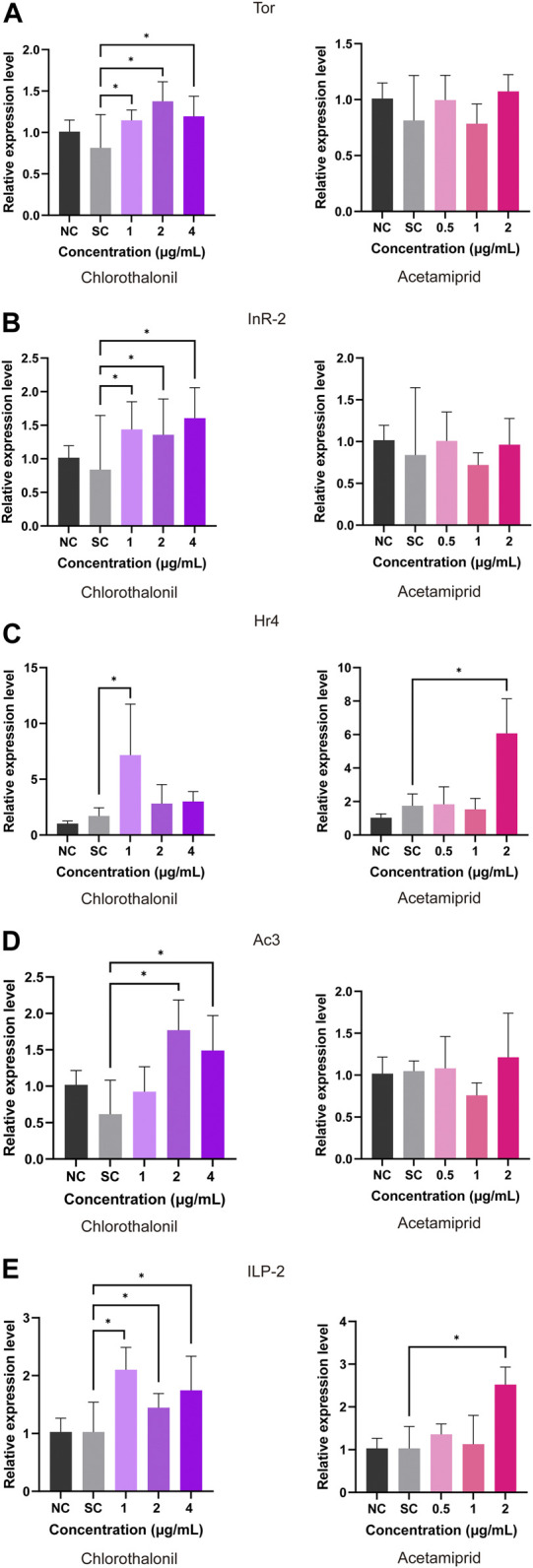
Effect of chlorothalonil and acetamiprid on the relative expression levels on *Tor* **(A)**, *AmInR-2* **(B)**, *Hr4* **(C)**, *Ac3* **(D)** and *ILP-2* **(E)** genes in *A. mellifera* larvae. After exposed to 1, 2 and 4 μg/mL chlorothalonil or 0.5, 1 and 2 μg/mL acetamiprid for 4 days, larvae were collected and total RNA was extracted. Each sample was assayed 4 times. Expression levels were normalized to *actin* and then to the gene expression level of the solvent control (SC). Significant differences were set at * for *p* < 0.05.

The relative expression of division related genes in honey bee larvae were quantified. As shown in Figure 2, the *Tor* and *AmInR-2* transcripts were upregulated upon exposure to 1, 2 and 4 μg/mL chlorothalonil. The expression of *Hr4* was upregulated at 4.1 folds and 3.5 folds at 1 μg/mL chlorothalonil and 2 μg/mL acetamiprid, but this change did not occur after treatment with 2 and 4 μg/mL chlorothalonil (Figure 2C). The transcript of *Ac3* was strong upregulated at 2 and 4 μg/mL chlorothalonil (Figure 2D). Acetamiprid induced the expression of *ILP-2* at 2 μg/mL, but did not alter the abundance of the other transcripts. And the *ILP-2* transcript was upregulated after expose to 1, 2, 4 μg/mL chlorothalonil (Figure 2E). When the concentration of acetamiprid was less than 2 μg/mL, the expression of all division related genes we tested was not significantly affected.

### Influence of acetamiprid and chlorothalonil on the expression of immune related genes

**FIGURE 3:**
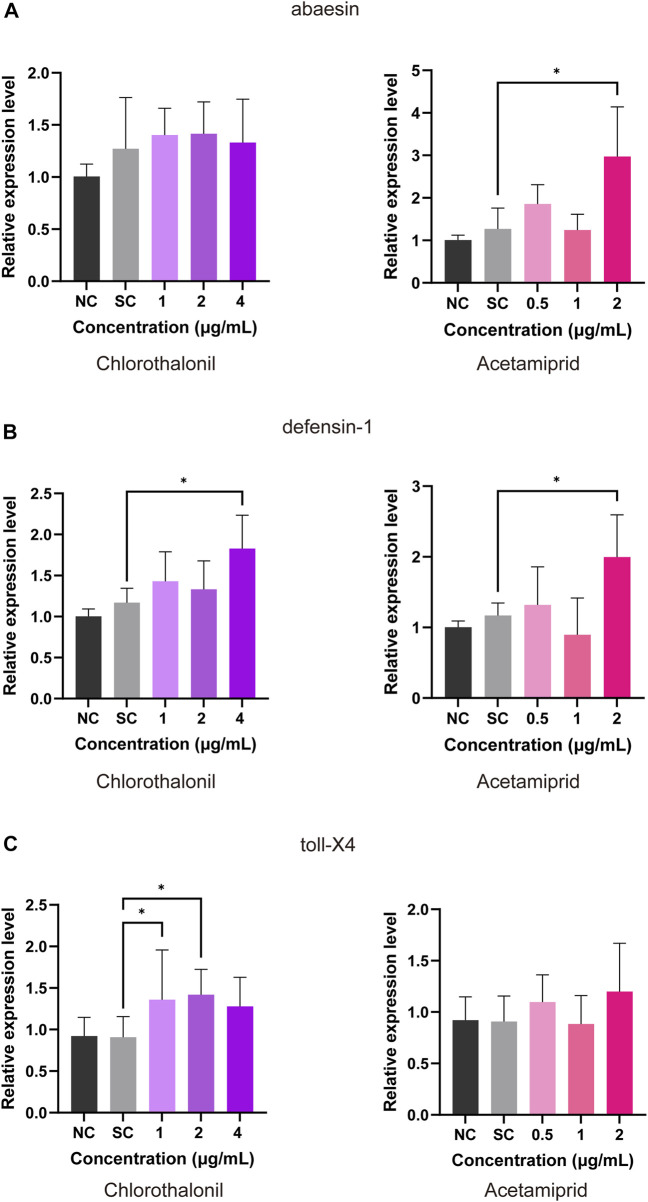
Effect of chlorothalonil and acetamiprid on the relative expression levels on *abaecin* **(A)**, *defensin* **(B)** and *toll-X4* **(C)** genes in *A. mellifera* larvae. After exposed to 1, 2 and 4 μg/mL chlorothalonil or 0.5, 1 and 2 μg/mL acetamiprid for 4 days, larvae were collected and total RNA was extracted. Each sample was assayed 4 times. Expression levels were normalized to *actin* and then to the gene expression level of the solvent control (SC). Significant differences were set at * for *p* < 0.05.

The transcript of *abaecin* was significantly upregulated at 2 μg/mL acetamiprid but did not remarkably affected by chlorothalonil (Figure 3A). In comparison to the solvent control, the expression of *defensin* was facilitated by 2 μg/mL acetamiprid or 4 μg/mL chlorothalonil (Figure 3B). Exposure to chlorothalonil resulted in upregulation of the *toll-X4* transcript at 1 and 2 μg/mL chlorothalonil, while acetamiprid had no effects on the expression of these transcripts (Figure 3C). But when the concentration of chlorothalonil reached 4 μg/mL, the expression of *toll-X4* was downregulated to the control value (Figure 3C).

### Influence of acetamiprid and chlorothalonil on the expression of detoxification related genes

**FIGURE 4:**
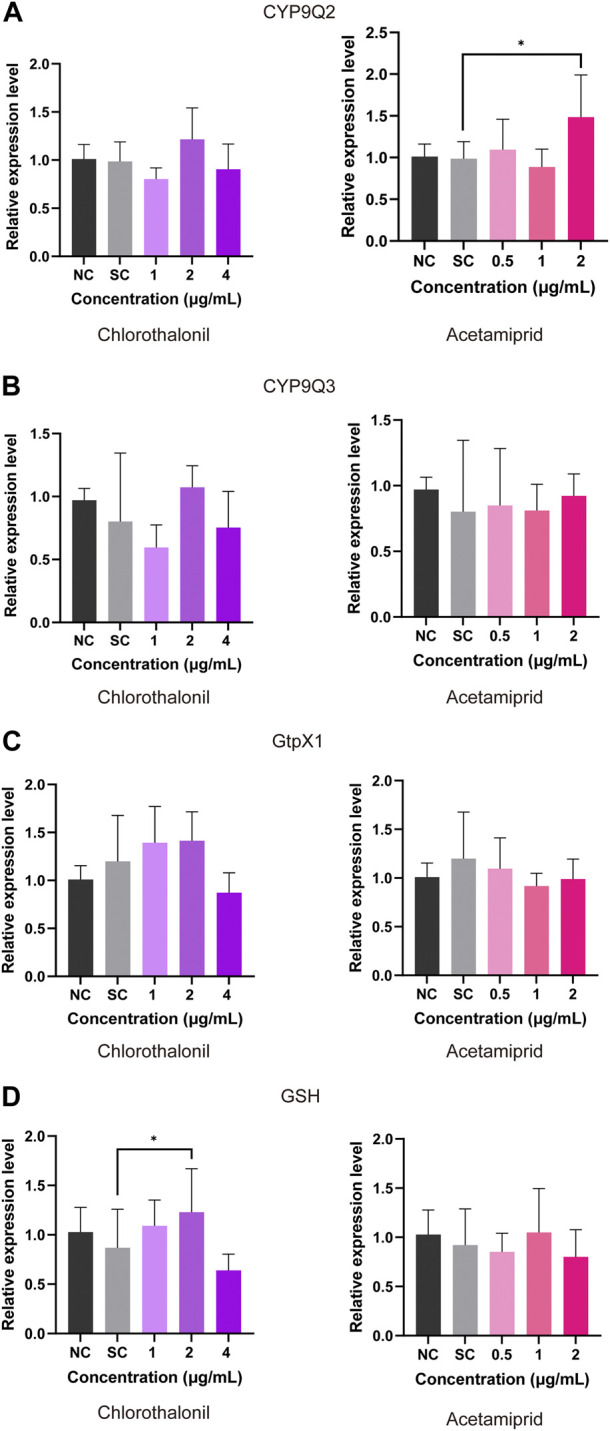
Effect of chlorothalonil and acetamiprid on the relative expression levels on *CYP9Q2* **(A)**, *CYP9Q3* **(B)**, *GtpX1* **(C)** and *GSH* **(D)** genes in *A. mellifera* larvae. After exposed to 1, 2 and 4 μg/mL chlorothalonil or 0.5, 1 and 2 μg/mL acetamiprid for 4 days, larvae were collected and total RNA was extracted. Each sample was assayed 4 times. Expression levels were normalized to *actin* and then to the gene expression level of the Solvent Control (SC). Significant differences were set at * for *p* < 0.05.

In comparison to the solvent control, the expression of *CYP9Q2* was upregulated after expose to 2 μg/mL acetamiprid (Figure 4A). No significant changes of the *CYP9Q3* transcript occurred in the treatment of chlorothalonil and acetamiprid (Figure 4B). Exposure to both acetamiprid and chlorothalonil also had no significant effects on the expression of *GtpX1* (Figure 4C). In addition, the transcript of *GSH* was significantly upregulated at 2 μg/mL chlorothalonil, but had no significant difference compared to the control at 4 μg/mL chlorothalonil (Figure 4D).

### The effect of chlorothalonil and acetamiprid on detoxification enzymes in larvae

**FIGURE 5:**
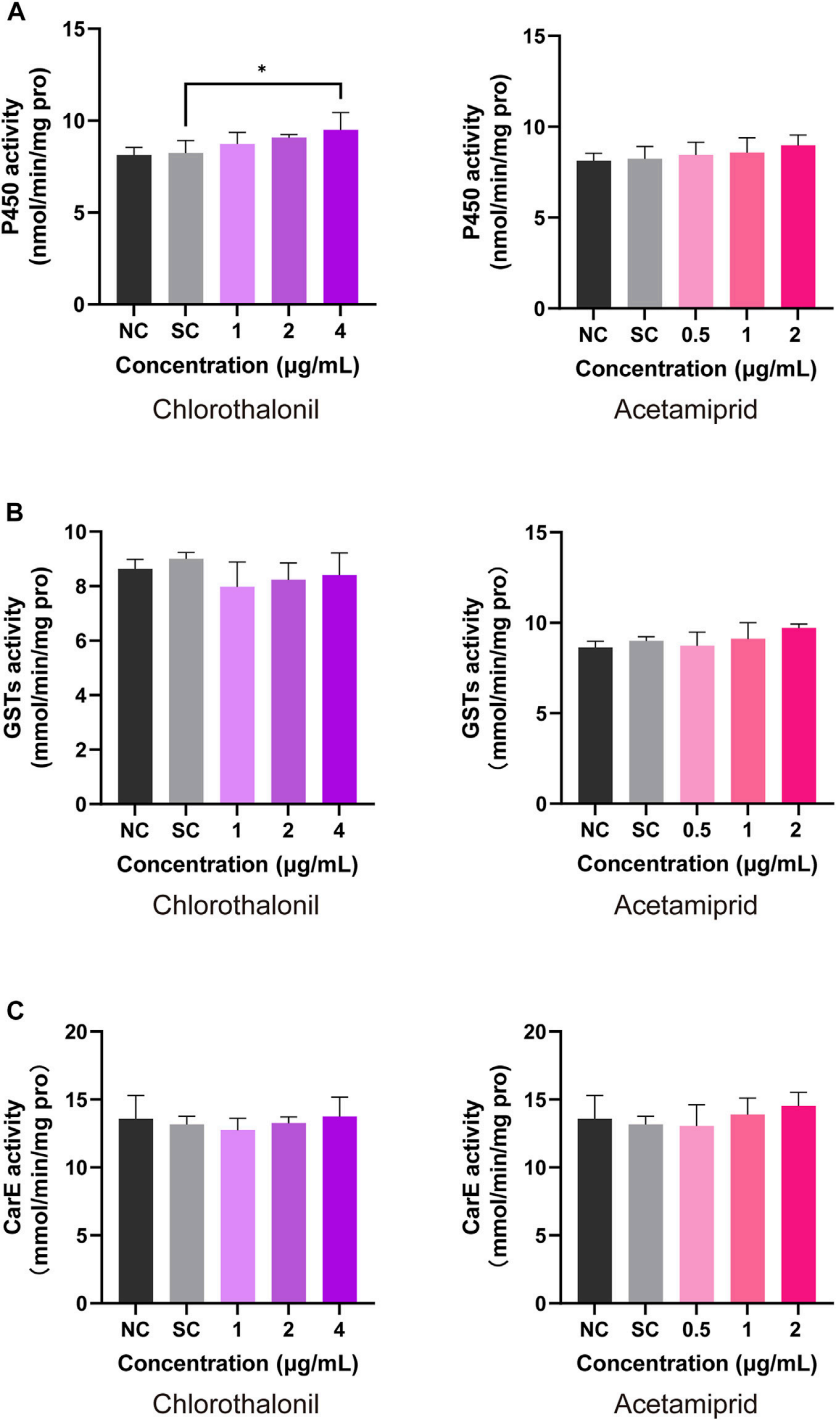
Response of honey bee larval P450 **(A)**, GST **(B)** and CarE **(C)** activity to chlorothalonil and acetamiprid. After exposed to 1, 2 and 4 μg/mL chlorothalonil or 0.5, 1 and 2 μg/mL acetamiprid for 4 days, larvae were collected. Each sample was assayed 3 times. Significant differences were set at * for *p* < 0.05.

After continuous intake of the diet contained chlorothalonil or acetamiprid, significant variation detoxification enzyme activity was identified in honey bee larvae. As seen in Figure 5A, the activity of P450 was significantly increased in the treatment of 2 μg/mL acetamiprid and 4 μg/mL chlorothalonil. The P450 activity of solvent control was 8.25 ± 0.67 nmol/min/mg pro and increased to 9.50 ± 0.94 nmol/min/mg pro, after exposed to 4 μg/mL chlorothalonil. But the difference of GSTs activity between solvent control and all treatments were not significant (Figure 5B). In addition, acetamiprid and chlorothalonil also had no significant effects on the activity of CarE in honey bee larvae (Figure 5C).

## Discussion

Previous research shows that exposure of honey bees to pesticides during larval stages can cause significant hidden harm to colonies (Davis et al., 1988; Davis, 1989). Broad spectrum fungicides and neonicotinoids are commonly used in agriculture and have been found in bee bread, beeswax and foraged environment (McAfee et al., 2021). Although not acutely toxic to bees, several studies have reported potential sublethal effects of chlorothalonil and acetamiprid, particularly in larvae (Wagner et al., 2021; Wu et al., 2022). In this study, we investigated the chronic toxicity of chlorothalonil and acetamiprid on developing larvae. Further, we tested the gene expression and enzyme activity to further demonstrate whether honey bee larvae were adversely affected in terms of immune and detoxification responses when exposed to the two pesticides at the level where no adverse effects were observed.

The chronic toxicity results indicated that the NOAEC of chlorothalonil and acetamiprid were 4 μg/mL and 2 μg/mL (Figure 1), respectively, which were lower than those reported in previous studies (Dai et al., 2018; Yang et al., 2020). This is not surprising since colonies of the same bee species with different genetic resources may have different tolerances to chemical xenobiotics (Johnson, 2015). In addition, the NOAEC of chlorothalonil and acetamiprid were much lower than the median residual level in pollen and nectar, suggesting that developing bee larvae are posing high risks of exposure to these insecticides (Mullin et al., 2010).

Numerous studies have been suggested that exposure to agricultural products have negative effects on growth, development and worker division of labor in honey bee colonies in honey bee (Ma et al., 2016; Tesovnik et al., 2020; Li et al., 2022). In the present study, we determined the transcriptional levels of *Tor* (GB44905), *AmInR-2* (GB55425), *Hr4* (GB17681), *Ac3* (GB11637) and *ILP-2* (GB10174), which are parts of the Insulin/insulin-like (ILP) and target of rapamycin (TOR) nutrient signaling pathway involved in the regulation of queen/worker caste development in honeybees (Wheeler et al., 2006; de Azevedo and Hartfelder, 2008). We observed that the expressions of the test genes were all upregulated in larvae exposed to chlorothalonil, although the exposure dose was lower than NOAEC. However, these changes were not shown in the acetamiprid-exposed groups treated at concentrations lower than NOAEC in this study (Figure 2). The overexpression of insulin-like growth factor signaling pathway related genes implies the increased nutrient storage. As one of the key regulators in energy metabolism, the insulin/TOR pathway also related to xenobiotics metabolism and stress resistance (Siede et al., 2012; du Rand et al., 2017; Gao et al., 2022). Continuously exposure to organic insecticide could contribute to insulin resistance and metabolic disorders in mammal and insects (de Azevedo and Hartfelder, 2008; Zhang et al., 2021). It has been reported that dietary nicotine stimulated an increase in the synthesis of energy by upregulated two proteins involved in the Insulin/Insulin-like growth factor signaling pathway (IIS) in honey bee larvae (du Rand et al., 2017). The upregulation of genes in this pathway implies that honeybee larvae cope with long-term insecticide exposure by energetically compensating for the adaptive cost of maintaining a highly resistant phenotype. Whether the low doses of chlorothalonil could promote the growth of honey bee larvae need further evidence for validation.

Sublethal concentration of insecticides are known to induce transcriptional changes associated with honeybee immune (Evans et al., 2006; Randolt et al., 2008; Shi et al., 2020). Antimicrobial peptides and protein toll are essential components of the Toll-like receptor pathway, which have been identified in the researches for innate immunity in honey bee (Chan et al., 2009; Danihlík et al., 2015; Horak et al., 2020; Bartling et al., 2021). According to our data, antimicrobial peptides including *abaecin* (GB18323) and *defensin-1* (GB19392) were significantly upregulated in larvae exposed to acetamiprid at NOAEC. Exposure of larvae to NOAEC of chlorothalonil increased the expression of *defensin-1* (GB19392) and *toll-X4* (GB50418), but not *abaecin* (GB18323) (Figure 3). Exposure to concentrations below the NOAED did not cause transcriptional changes, reflecting a dose-effect relationship (Figure 3). The antimicrobial peptides (AMPs) response to xenobiotic toxicity or pathogens disease *via* the rapid expression (Bulet et al., 1999). *Defensin-1* (GB19392) is expressed in the salivary glands and is effective against both G+ and G-bacteria, whereas abaecin is less active against most G-bacteria (Klaudiny et al., 2005). The protein toll was originally shown to be a type 1 transmembrane receptor that controls dorsal-ventral patterning and later was identified to be involved in host resistance against pathogens (Jang et al., 2006). The upregulation of these genes can lead to immunity activation for improving the resistance to toxin.

We also assayed the mRNA expression levels and activities of enzymic biomarkers in larvae exposed to different concentrations of chlorothalonil and acetamiprid, respectively. As shown in Figure 4, the expression of three detoxification-related genes (*CYP9Q3*, *GtpX1*, *GSH*) had no significant difference between the acetamiprid-exposed and control groups. In addition, the enzymic activities of P450, GST and CarE was also not influenced by acetamiprid (Figure 5). Our findings were consistent with previous studies where exposure to low doses of acetamiprid had very limited effects on molecular disturbances in worker larvae (Wright et al., 2015; Gao et al., 2020). The most probable explanation is that low dose of acetamiprid can be quickly distributed and detoxified in larval, resulting in relatively low toxicity (Iwasa et al., 2004; Brunet et al., 2005; Yang et al., 2020). Cytochrome P450s is a major detoxification enzyme superfamily involved in metabolic detoxification and resistance of many insecticides (Johnson et al., 2012; Katsavou et al., 2022). *CYP9Q2* (GB17793) and *CYP9Q3* (GB19967), members in clade CYP9, were most frequently involved in xenobiotic metabolism and evolution of the hormonal and chemosensory processes in *A. mellifera* and *Bombus terrestris* (Claudianos et al., 2006; Manjon et al., 2018). In the present study, we found that only *CYP9Q2* (GB17793) was upregulated in larvae after exposed to acetamiprid for 4 days, suggesting that honey bees activate the expression of specific isoforms to response to chemical toxicity. When pesticides induced oxidative stress in honey bee, reactive oxygen species (ROS) are generated with P450-mediated detoxification processes. Glutaredoxins act as immediate electron donor and its contribution to providing resistance to oxidative stress during honeybee detoxification processes has been well described (Xu et al., 2013; Yao et al., 2014; Orčić et al., 2022).

The relative expression of *glutaredoxin-C4* (GSH) was significantly increased in bees exposed to 2 μg/mL chlorothalonil (Figure 4D). Moreover, the P450 activity was slightly induced by 4 μg/mL chlorothalonil (Figure 5A). Our findings are in line with previous statement that these antioxidant proteins can be temporarily overproduced under low-dose pesticide stress (du Rand et al., 2017; Pal et al., 2022). However, at 4 μg/mL chlorothalonil, the transcript of *GSH* was not significantly upregulated (Figure 4D). The result implied that the transcript levels of *GSH* are not different from control, but the GSH cycle has been broken by 4 μg/mL chlorothalonil (Qi et al., 2020).

## Conclusion

With the wide use of synthetic organic pesticides, the fungicides and neonicotinoids are posing potential risks to honey bees larvae. By testing the transcriptional levels and activities of major enzymic biomarkers, the risk of chronic exposure of acetamiprid at NOAEC to bee larvae were acceptable. However, the exposure to chlorothalonil, even at concentrations below the NOAEC, had potential effects on honey bee larvae. Thus, the effects of chlorothalonil at no observed adverse effect concentration on colony fitness should be explored in the future, and should be given due consideration for its application in crops pollinated and visited by honey bees.

## Data Availability

The original contributions presented in the study are included in the article/Supplementary Material, further inquiries can be directed to the corresponding authors.

## Abbreviations

Ac3, Adenylate cyclase 3; AmInR-2, *Apis mellifera* insulin-like receptor-like 2; CarE, Carboxylesterase; CYP, Cytochrome P450; CYP9Q2, Cytochrome P450 9Q2; CYP9Q3, Cytochrome P450 9Q3; GSH, Glutaredoxin-C4; GSTs, Glutathione S-transferases; GtpX1, Glutathione peroxidase-like 1; Hr4, Hormone receptor 4; ILP-2, Insulin-like peptide 2; NOAEC, No observed adverse effect concentration; Tor, Target of rapamycin.
